# Assessing the continuum of care for maternal health in Mexico, 1994–2018

**DOI:** 10.2471/BLT.20.252544

**Published:** 2020-11-30

**Authors:** Edson Serván-Mori, Ileana Heredia-Pi, Diego Cerecero García, Gustavo Nigenda, Sandra G Sosa-Rubí, Jacqueline A Seiglie, Rafael Lozano

**Affiliations:** a Center for Health System Research, National Institute of Public Health, Avenida Universidad #655, 62100, Cuernavaca, Morelos, Mexico.; b National School of Nursing and Obstetrics, National Autonomous University of Mexico, Mexico City, Mexico.; c Diabetes Unit, Massachusetts General Hospital, Harvard Medical School, Boston, United States of America (USA).; d Institute for Health Metrics and Evaluation, University of Washington, Seattle, USA.

## Abstract

**Objective:**

To describe the temporal and geographical patterns of the continuum of maternal health care in Mexico, as well as the sociodemographic characteristics that affect the likelihood of receiving this care.

**Methods:**

We conducted a pooled cross-sectional analysis using the 1997, 2009, 2014 and 2018 waves of the National Survey of Demographic Dynamics, collating sociodemographic and obstetric characteristics of 93 745 women aged 12–54 years at last delivery. We defined eight variables along the antenatal–postnatal continuum, both independently and conditionally. We used a pooled fixed-effects multivariable logistic model to determine the likelihood of receiving the continuum of care for various properties. We also mapped the quintiles of adjusted state-level absolute change in continuum of care coverage during 1994–2018.

**Findings:**

We observed large absolute increases in the proportion of women receiving timely antenatal and postnatal care (from 48.9% to 88.2% and from 39.1% to 68.7%, respectively). In our conditional analysis, we found that the proportion of women receiving adequate antenatal care doubled over this period. We showed that having social security and a higher level of education is positively associated with receiving the continuum of care. We observed the largest relative increases in continuum of care coverage in Chiapas (181.5%) and Durango (160.6%), assigned human development index categories of low and medium, respectively.

**Conclusion:**

Despite significant progress in coverage of the continuum of maternal health care, disparities remain. While ensuring progress towards achievement of the health-related sustainable development goal, government intervention must also target underserved populations.

## Résumé

**Objectif:**

Décrire les schémas géographiques et temporels de la continuité des soins de santé maternelle au Mexique, ainsi que les caractéristiques sociodémographiques qui déterminent la probabilité de recevoir de tels soins.

**Méthodes:**

Nous avons effectué une analyse transversale combinée fondée sur les éditions 1997, 2009, 2014 et 2018 de l'Enquête nationale sur la dynamique démographique, et compilé les données sociodémographiques et obstétriques de 93 745 femmes âgées de 12 à 54 ans lors de leur dernier accouchement. Nous avons identifié huit variables au fil de la continuité prénatale et postnatale, tant de manière indépendante que conditionnelle. Puis nous avons employé un modèle logistique multivarié à effets fixes combinés pour déterminer la probabilité de bénéficier de cette continuité des soins pour diverses propriétés. Enfin, nous avons cartographié les quintiles d'écart absolu ajusté au niveau national dans la continuité de la couverture durant la période comprise entre 1994 et 2018.

**Résultats:**

Nous avons observé d'importantes augmentations réelles dans le pourcentage de femmes faisant l'objet de soins prénatals et postnatals au moment opportun (de 48,9% à 88,2% et de 39,1% à 68,7% respectivement). Notre analyse conditionnelle a révélé que la proportion de femmes ayant reçu des soins prénatals adaptés a doublé au cours de cette période. Nous avons démontré que le fait d'avoir une sécurité sociale et un niveau d'éducation plus élevé était associé à une amélioration de l'accès à la continuité des soins. Ce sont les États de Chiapas (181,5%) et de Durango (160,6%) qui ont connu les plus grandes augmentations relatives en matière de continuité des soins, le premier appartenant à la catégorie faible de l'indice de développement humain, et le second à la catégorie moyenne.

**Conclusion:**

Malgré les progrès considérables réalisés dans la couverture d'une continuité des soins de santé maternelle, des inégalités subsistent. Le gouvernement doit continuer à tendre vers l'objectif de développement durable lié à la santé tout en focalisant son intervention sur les populations mal desservies.

## Resumen

**Objetivo:**

Describir los patrones temporales y geográficos de la continuidad de la atención sanitaria materna en México, así como las características sociodemográficas que afectan a la probabilidad de recibir esta atención.

**Métodos:**

Realizamos un análisis transversal conjunto utilizando las curvas de 1997, 2009, 2014 y 2018 de la Encuesta Nacional de la Dinámica Demográfica (ENADID), cotejando las características sociodemográficas y obstétricas de 93.745 mujeres de 12 a 54 años en el último parto. Definimos ocho variables a lo largo de la continuidad prenatal-postnatal, tanto de forma independiente como condicionada. Utilizamos un modelo logístico multivariable de efectos fijos combinados para determinar la probabilidad de recibir la continuidad de la atención para diversas propiedades. También trazamos un mapa de los quintiles de cambio absoluto ajustado a nivel estatal en la cobertura de la continuidad de la atención durante 1994-2018.

**Resultados:**

Observamos grandes aumentos absolutos en la proporción de mujeres que reciben atención prenatal y postnatal oportuna (del 48,9% al 88,2% y del 39,1% al 68,7%, respectivamente). En nuestro análisis condicional, encontramos que la proporción de mujeres que reciben atención prenatal adecuada se duplicó durante este período. Demostramos que tener seguridad social y un mayor nivel de educación está positivamente asociado con la recepción de la atención continua. Observamos los mayores incrementos relativos en la cobertura de la continuidad de atención en Chiapas (181,5%) y Durango (160,6%), a los que se asignaron las categorías de índice de desarrollo humano de bajo y medio, respectivamente.

**Conclusión:**

A pesar de los importantes progresos realizados en la cobertura de la continuidad de la atención sanitaria materna, siguen existiendo disparidades. Al tiempo que se garantiza el progreso hacia el logro del objetivo de desarrollo sostenible relacionado con la salud, la intervención del gobierno también debe dirigirse a las poblaciones desatendidas.

## ملخص

الغرض وصف الأنماط الزمنية والجغرافية لاستمرارية الرعاية الصحية للأم في المكسيك، فضلاً عن الخصائص الاجتماعية السكانية التي تؤثر على احتمالية تلقي هذه الرعاية.

الطريقة قمنا بإجراء تحليل مقطعي مجمع باستخدام موجات من المسح الوطني للديناميكيات السكانية في أعوام 1997 و2009 و2014 و2018، وجمع الخصائص الاجتماعية السكانية والخصائص التوليدية لعدد 93745 امرأة تتراوح أعمارهن بين 12 و54 عاماً عند آخر عملية ولادة. قمنا بتحديد ثمانية متغيرات بامتداد الفترة المتصلة قبل الولادة وبعدها، بشكل مستقل ومشروط. ثم استخدمنا نموذج لوجيستي متعددة المتغيرات ذي تأثيرات محددة ومجمعة، لتحديد احتمالية الحصول على استمرارية الرعاية لمختلف الظروف. قمنا أيضًا بتعيين فئات خمسية للتغير المطلق المعدل على مستوى الولاية في استمرار تغطية الرعاية خلال الفترة من 1994 إلى 2018.

النتائج لاحظنا زيادات مطلقة كبيرة في نسبة النساء اللواتي يحصلن على الرعاية قبل الولادة وبعدها في الوقت المناسب (من 48.9% إلى 88.2%، ومن 39.1% إلى 68.7% على الترتيب). وفي هذا التحليل الشرطي، وجدنا أن نسبة النساء اللواتي يحصلن على رعاية مناسبة قبل الولادة، قد تضاعفت خلال هذه الفترة. لقد أوضحنا أن الحصول على الأمان الاجتماعي ومستوى أعلى من التعليم، يرتبط بشكل إيجابي بالحصول على رعاية مستمرة. لاحظنا أن أكبر الزيادات النسبية في استمرارية تغطية الرعاية في تشياباس (181.5%)، ودورانجو (160.6%)، كانت حيث تم تعيين فئات مؤشر التنمية البشرية بأنها منخفضة ومتوسطة، على الترتيب.

الاستنتاج على الرغم من التقدم الملموس في تغطية الرعاية الصحية المستمرة للأم، إلا أنه لا تزال هناك فوارق قائمة. مع ضمان التقدم نحو تحقيق هدف التنمية المستدامة المتعلق بالصحة، يجب أن يستهدف التدخل الحكومي أيضاً الفئات السكانية المحرومة من الخدمات.

## 摘要

**目的:**

旨在描述墨西哥孕产妇连续保健服务在时间和地理层面的分布状况，以及影响享受这种保健服务的社会人口学特征。

**方法:**

我们采用 1997 年、2009 年、2014 年和 2018 年全国人口动态调查的数据开展了横断面调查汇总分析，并整理了 93,745 名年龄介于 12 至 54 岁之间的妇女上一次分娩时的社会人口学和产科特征。我们采用独立和条件分析围绕产前–产后连续性定义了八个变量。我们运用汇总的固定效应多元 Logistic 回归模型来确定各属性接受连续护理的可能性。同时还划分了 1994 年至 2018 年期间连续护理覆盖率调整后的国家层面变化绝对值的五分位点。

**结果:**

我们发现，及时接受产前和产后护理的妇女比例大幅增加（分别从 48.9% 增至 88.2% 以及从 39.1% 增至 68.7%）。根据我们的条件分析，我们发现，在此期间充分接受产前护理的孕妇比例翻了一番。分析显示，拥有社会保障和较高教育水平与获得连续护理呈正相关。根据我们的观察，恰帕斯 (181.5%) 和杜兰戈 (160.6%) 的连续护理覆盖率相对增幅最大，分别属于较低和中等人类发展指数类别。

**结论:**

尽管孕产妇连续保健服务的覆盖率取得了显著进展，但仍存在明显差异。确保在实现健康相关可持续发展目标方面取得进展的同时，政府还必须针对服务匮乏地区的人口采取干预措施。

## Резюме

**Цель:**

Описать временные и географические закономерности системы бесперебойного и непрерывного оказания помощи матерям в Мексике, а также социально-демографические характеристики, которые влияют на вероятность получения таких услуг.

**Методы:**

Авторы провели объединенный перекрестный анализ с использованием волн Национального обследования демографической динамики за 1997, 2009, 2014 и 2018 годы, сопоставив социально-демографические и акушерские характеристики для 93 745 женщин в возрасте от 12 до 54 лет на момент последних родов. Были определены восемь переменных в системе бесперебойного и непрерывного оказания помощи в дородовой и послеродовой период, как независимых, так и условных. Авторы использовали объединенную многомерную логистическую модель с фиксированными эффектами, чтобы определить вероятность бесперебойного и непрерывного оказания помощи для различных свойств. На уровне отдельных штатов была также составлена карта квинтилей скорректированных абсолютных изменений в системе бесперебойного и непрерывного оказания помощи за период 1994–2018 гг.

**Результаты:**

Авторы наблюдали значительный абсолютный прирост доли женщин, получающих своевременную дородовую и послеродовую помощь (с 48,9 до 88,2% и с 39,1 до 68,7% соответственно). В своем условном анализе авторы обнаружили, что доля женщин, получающих надлежащую дородовую помощь, за этот период удвоилась. Показано, что наличие социального обеспечения и более высокий уровень образования положительно связаны с получением бесперебойной и непрерывной помощи. Наибольшее относительное увеличение охвата услугами системы бесперебойного и непрерывного оказания медицинской помощи наблюдалось в штатах Чьяпас (181,5%) и Дуранго (160,6%), которым были присвоены категории низкого и среднего индекса гуманитарного развития соответственно.

**Вывод:**

Несмотря на значительный прогресс в системе бесперебойного и непрерывного оказания помощи матерям, неравенство все еще сохраняется. Наряду с обеспечением прогресса в достижении цели в области устойчивого развития, связанной со здоровьем, вмешательство государства также должно быть направлено и на малообеспеченные слои населения.

## Introduction

Despite significant progress in the provision of maternal health care in low- and middle-income countries as a result of the millennium and sustainable developments goals (SDGs),^1^ significant challenges in the provision of both maternal and universal health coverage (UHC) remain. Gaps in the coverage of important maternal health interventions^2^^–^^5^ that are closely associated with social vulnerability have also been identified.^6^^–^^11^

Effective policies to improve health outcomes and promote the full implementation of UHC require changes in procedures from monitoring crude coverage to quality-adjusted coverage.^12^ Recent studies have suggested that current methods of measuring intervention coverage for reproductive and maternal health do not adequately determine the quality of services delivered; without information on the quality of care, it is difficult to assess expected health improvements.^13^^,^^14^ These recent studies have also quantified the alarming discrepancies between the impact on women’s health as measured from crude coverage indicators and the impact as measured from contact coverage indicators (i.e. that represent the delivery and benefits from high-quality services).^12^

Widely accepted as a proxy for quality-adjusted coverage indicators, the continuum of care principle for maternal, newborn and child health aims to reduce the burden of maternal and child mortality by integrating health services throughout the life cycle.^15^^–^^18^ According to Kerber’s framework, the continuum of care has two dimensions:^15^ (i) time, which refers to the linking of health care during adolescence and pre-pregnancy through childbirth, the immediate postnatal period and childhood; and (ii) place, which refers to the linking of health care that is provided across different environments, including households, communities and clinical care at different levels.^15^^,^^19^^,^^20^ The continuum of care therefore aims to provide women with reproductive health services and newborns with the opportunity of a healthy childhood,^15^^,^^20^ but also to ensure that services are delivered in an integrated way to avoid inefficiency, control associated costs,^15^^,^^20^ and minimize maternal and neonatal mortality.^16^

Over the past 25 years,^21^ the priorities of maternal, newborn and child health have emerged as key within the Mexican health-care system, and financial protection was provided in the form of the *Seguro Popular de Salud*. The now-obsolete government-financed *Seguro Popular de Salud*, a voluntary family health insurance programme for those without social security (i.e. the self-employed, underemployed and unemployed), was operational in 2003^22^^–^^24^ until it was replaced by the Health Institute for Wellbeing by a new administration in early 2020. To inform health-care policies as we work towards the 2030 target of the SDGs, we need a comprehensive assessment of progress made and challenges remaining in monitoring the quality-adjusted coverage of maternal health care. Our aims are therefore to: (i) describe the temporal and geographical trends in the provision of the continuum of maternal health care in Mexico during the past 25 years; and (ii) determine the sociodemographic and obstetric characteristics that affect the likelihood of this continuum of care being received.

## Methods

### Study design

We conducted a pooled cross-sectional analysis using the 1997, 2009, 2014 and 2018 waves of the National Survey of Demographic Dynamics.^25^ Implemented by the National Institute of Statistics and Geography of Mexico, these cross-sectional, probabilistic, retrospective population-based surveys are representative at both the national and state level and across different residential areas.^25^ The four surveys include the sociodemographic and reproductive characteristics of 98 156 women aged 12–54 years at the time of last delivery. After excluding 4.5% of participants who did not provide complete survey responses, our survey population included 93 745 women. A comparison of the sociodemographic and health-related characteristics between women who were included in and excluded from our survey population found no statistically significant differences.

We obtained data on the sociodemographic characteristics of the survey participants at an individual and place of residence or contextual level, including age at time of last delivery, whether at least one indigenous language spoken, marital status, level of education, whether recently employed and health insurance status at the time of the survey. We also recorded obstetric information, such as: whether primiparous; whether the woman had experienced an infant death, miscarriage or abortion, or a health problem during pregnancy or childbirth; and type of delivery. At the household level, we included a factorial asset and housing material index as a measure of socioeconomic status. We used this index to stratify participants over five categories according to the method of Dalenius and Hodges,^26^ where the higher categories indicate a greater number of assets and better housing conditions. We classified type of residence as either rural (< 2500 inhabitants), urban (2500–100 000 inhabitants) or metropolitan (> 100 000 inhabitants).

### Continuum of maternal health care 

Our approach focuses on the routine processes that are recommended during contact between mother and health-care provider. However, we defined our main outcome variable as the quality-adjusted conditional coverage indicator, a measure of the receipt of high-quality services and not simply contact with a health-care provider.^12^

First, we defined our eight independent coverage indicators of the continuum of care in terms of antenatal and postnatal health-care processes, that is, whether: (i) antenatal care was received; (ii) antenatal care was provided by a skilled birth attendant (doctor or nurse); (iii) the first medical visit occurred during the first 8 weeks of pregnancy (timely antenatal care); (iv) at least five antenatal consultations were received (frequent antenatal care); (v) antenatal care included at least 75% of recommended care^17^^,^^27^ (adequate antenatal care; defined as receipt of 60–80% of recommended care elsewhere^28^^,^^29^); (vi) the delivery was attended by skilled personnel; (vii) a postnatal consultation was received; and (viii) postnatal care occurred within 15 days after delivery (timely postnatal care), according to Mexican health-care system guidelines^30^ and the outcomes of our previous research.^17^^,^^31^

We define all coverage indicators according to international recommendations made by the World Health Organization (WHO),^32^^,^^33^ with some minor exceptions. The WHO guidelines suggest a minimum of eight antenatal care visits,^32^^,^^33^ whereas Mexican health-care system guidelines recommend at least five visits.^30^ Additionally, Mexican guidelines recommend that the first prenatal care visit takes place during gestational weeks 6–8, whereas WHO references the first trimester.^32^^,^^33^ Similarly, the number of postnatal appointments recommended nationally are a minimum of two clinic visits, one within 15 days of the birth and the second at the end of the puerperium.^30^ In contrast, the WHO guidelines recommend three visits over the same time period.^32^^,^^33^ Worth noting is that the definition of what is included within recommended antenatal care has changed in Mexico during the 25-year study period. An abdominal examination was performed in the 2009 survey only, while mother’s weight measurement was excluded from the 2014 survey. The 2009, 2014 and 2018 surveys included ultrasound, blood and urine tests, the prescription of vitamins and/or mineral supplementation, and human immunodeficiency virus testing. The 2018 survey included height measurement, data on fetal movement and mental health services.

We defined a further eight binary outcome variables indicating the incremental access to interventions (i)–(viii) along the antenatal–postnatal continuum. We constructed these conditional coverage indicators using the coverage cascade principle, in which receiving the care described by each separate independent indicator is conditional on receiving the care described by the preceding independent indicator.^12^ We defined the proportion of women who were considered to have received a continuum of care as the proportion who received all eight antenatal–postnatal interventions.

### Statistical analysis

We performed all analyses using the “svy” module and sampling weights of the statistical software Stata version 15.1 (StataCorp, College Station, United States of America). We calculated the sociodemographic and obstetric characteristics of the 93 745 surveyed women according to the period of last delivery (1994–1997, 2004–2009, 2010–2014 and 2015–2018), then converted the survey data to weighted population-level estimates (population, 29 822 452) with 95% confidence intervals (CIs).^25^ We then converted these estimates to percentages of the relevant population receiving each of the individual health-care interventions. Our modelled conditional coverage indicators refer to compliance with all eight health-care interventions.

We used a pooled fixed-effects multivariable logistic model to determine which sociodemographic and obstetric characteristics affect the likelihood of receiving the continuum of maternal health care. We adjusted our model for all covariates recorded in the surveys (except for type of delivery, because of its temporality), including survey year and a binary variable for each state (i.e. state fixed effect). We reported adjusted odds ratios with their 95% CIs. We then adjusted the prevalence of receiving the continuum of care according to health insurance. We also mapped the quintiles of adjusted absolute change in receipt of the continuum of care between 1994 and 2018 at the individual state level. 

## Results

From the first study wave to the most recent, we observed a decrease in the proportion of women who were either married or cohabiting with their partner from 89.4% (95% CI: 88.7–90.0) in 1994–1997 to 83.0% (95% CI: 82.2–83.8) in 2015–2018 (Table 1). We also observed an increase in the proportion of women who were head of the household, from 4.8% (95% CI: 4.4–5.3) in 1994–1997 to 7.9% (95% CI: 7.4–8.4) in 2015–2018. Regarding health insurance coverage, the percentage of women who reported having social security decreased from 38.4% (95% CI: 36.1–40.7) in 1994–1997 to 32.1% (95% CI: 31.0–33.1) in 2015–2018. The percentage of women without health insurance decreased from 61.6% (95% CI: 59.3–63.9) in 1994–1997 to 13.3% (95% CI: 12.5–14.0) in 2015–2018; this decrease was accompanied by an increase in women with *Seguro Popular de Salud* from 30.7% (95% CI: 29.8–31.7) in 2004–2009 to 54.7% (95% CI: 53.5–55.8) in 2015–2018.

**Table 1:** Trends in sociodemographic and obstetric characteristics of women aged 12–54 years in assessment of continuum of maternal health care, Mexico, 1994–2018

Sociodemographic and obstetric characteristics^a^	Estimated percentage of weighted population (95% CI)			
	1994–1997 (*n* = 6 334 289)	2004–2009 (*n* = 8 424 843)	2010–2014 (*n* = 9 450 735)	2015–2018 (*n* = 5 612 585)
**Age at last delivery (years)**				
12–19	14.1 (13.4–14.7)	15.2 (14.6–15.9)	16.3 (15.7–16.9)	17.6 (16.8–18.3)
20–29	57.8 (56.5–59.0)	53.1 (52.2–54.1)	53.3 (52.5–54.0)	54.2 (53.2–55.2)
30–39	25.3 (24.3–26.4)	29.1 (28.3–30.0)	27.7 (27.0–28.4)	25.7 (24.8–26.6)
40–54	2.9 (2.5–3.2)	2.5 (2.2–2.8)	2.7 (2.5–3.0)	2.6 (2.2–2.9)
**Household head**	4.8 (4.4–5.3)	7.7 (7.3–8.1)	9.1 (8.7–9.6)	7.9 (7.4–8.4)
**Speaks at least one indigenous language**	9.0 (7.3–10.7)	6.6 (5.8–7.5)	7.1 (6.5–7.8)	7.4 (6.5–8.3)
**Marital status**				
Single	5.4 (5.0–5.9)	8.5 (7.9–9.0)	8.1 (7.7–8.6)	8.0 (7.4–8.5)
Married or cohabiting	89.4 (88.7–90.0)	83.2 (82.5–83.9)	82.2 (81.6–82.8)	83.0 (82.2–83.8)
Divorced, separated or widowed	5.2 (4.7–5.7)	8.4 (7.9–8.9)	9.6 (9.2–10.1)	9.0 (8.5–9.6)
**Education (years)**				
0–6 (none or elementary school)	50.4 (47.6–53.1)	29.6 (28.6–30.6)	21.4 (20.6–22.1)	15.8 (15.0–16.7)
7–9 (secondary school)	32.5 (30.8–34.2)	35.4 (34.5–36.3)	37.7 (36.9–38.5)	38.3 (37.3–39.3)
10–12 (high school)	9.0 (8.2–9.9)	22.7 (21.9–23.5)	24.8 (24.1–25.6)	27.7 (26.8–28.6)
13–24 (higher education)	8.1 (7.2–9.0)	12.3 (11.6–12.9)	16.1 (15.4–16.7)	18.2 (17.3–19.0)
**Employed in the last week**	35.1 (33.9–36.3)	34.0 (33.1–34.9)	36.8 (36.0–37.5)	35.9 (34.8–36.9)
**Health insurance**				
Social security	38.4 (36.1–40.7)	35.5 (34.5–36.6)	31.5 (30.8–32.3)	32.1 (31.0–33.1)
*Seguro Popular de Salud *	NA	30.7 (29.8–31.7)	54.0 (53.1–54.9)	54.7 (53.5–55.8)
None	61.6 (59.3–63.9)	33.7 (32.7–34.7)	14.5 (13.9–15.1)	13.3 (12.5–14.0)
**Obstetric characteristics**				
Primiparous	30.7 (29.6–31.9)	32.3 (31.4–33.2)	34.5 (33.8–35.3)	38.2 (37.2–39.1)
History of stillbirth or infant death	3.3 (2.9–3.7)	2.3 (2.1–2.6)	1.9 (1.7–2.1)	1.7 (1.4–2.0)
At least one miscarriage or abortion	13.1 (12.4–13.8)	12.3 (11.7–12.9)	13.0 (12.5–13.6)	13.3 (12.6–13.9)
Health problem diagnosed during pregnancy	68.6 (67.5–69.7)	61.1 (60.1–62.1)	64.4 (63.6–65.1)	66.8 (65.8–67.8)
Health problem diagnosed during childbirth	48.3 (47.2–49.4)	43.9 (42.9–44.9)	37.5 (36.8–38.3)	40.2 (39.1–41.2)
Delivery by caesarean section	28.1 (26.6–29.6)	43.4 (42.4–44.3)	45.8 (44.9–46.6)	45.6 (44.5–46.7)
**Socioeconomic status**				
Lowest	17.3 (15.1–19.5)	2.6 (2.2–3.1)	1.8 (1.5–2.0)	1.6 (1.3–1.9)
Low	15.1 (13.3–16.8)	6.6 (6.0–7.3)	6.7 (6.2–7.2)	5.2 (4.6–5.8)
Medium	20.2 (18.9–21.5)	19.2 (18.3–20.2)	22.3 (21.5–23.1)	22.0 (21.0–23.1)
High	10.7 (9.9–11.5)	20.0 (19.1–21.0)	19.0 (18.3–19.7)	18.9 (18.0–19.7)
Highest	36.8 (33.9–39.6)	51.4 (50.2–52.6)	50.2 (49.2–51.1)	52.3 (51.0–53.6)
**Area of residence^b^**				
Rural	28.4 (23.1–33.7)	24.8 (23.7–25.9)	27.9 (27.1–28.8)	28.7 (27.1–30.4)
Urban	28.1 (23.1–33.1)	31.4 (30.3–32.5)	31.4 (30.3–32.4)	30.6 (29.0–32.2)
Metropolitan	43.5 (39.0–48.0)	43.9 (42.9–44.9)	40.7 (39.8–41.6)	40.7 (39.2–42.2)

CI: confidence interval; NA: not applicable.

^a^ The data set includes women who have experienced at least one pregnancy.

^b^ We classified areas with less than 2500 inhabitants as rural, areas with 2500–100 000 inhabitants as urban and areas with more than 100 000 inhabitants as metropolitan.

Note: The *Seguro Popular de Salud* programme was not introduced until 2003.

We note that 48.3% (95% CI: 47.2–49.4) of women had a health problem during childbirth in 1994–1997; this proportion was reduced to 40.2% (95% CI: 39.1–41.2) in 2015–2018. We also observed an increase in the proportion of women who had given birth by caesarean section from 28.1% (95% CI: 26.6–29.6) in 1994–1997 to 45.6% (95% CI: 44.5–46.7) in 2015–2018 (Table 1). In terms of independent coverage indicators, we observed the largest increases over the 25-year period in the proportion of women receiving timely antenatal care and postnatal care. We calculated an increase in receipt of timely antenatal care from 48.9% (95% CI: 47.2–50.6) in 1994–1997 to 88.2% (95%: 87.5–88.8) in 2015–2018, and an increase in receipt of postnatal care from 39.1% (95% CI: 37.7–40.5) in 1994–1997 to 68.7% (95% CI: 67.7–69.7) in 2015–2018 (Table 2).

**Table 2:** Trends in independent and conditional coverage indicators in assessment of continuum of maternal health care, Mexico, 1994–2018

Coverage indicator	Estimated percentage of weighted population (95% CI)					Absolute (relative) increase in percentage from 1994–1997 to 2015–2018^a^
	1994–1997 (*n* = 6 334 289)	2004–2009 (*n* = 8 424 843)	2010–2014 (*n* = 9 450 735)	2015–2018 (*n* = 5 612 585)		
**Independent **						
Received antenatal care	92.9 (92.0–93.9)	98.8 (98.6–99.0)	99.0 (98.9–99.2)	99.0 (98.8–99.2)		6.1 (6.6)
Skilled antenatal care	87.1 (85.6–88.6)	97.2 (96.8–97.6)	98.0 (97.8–98.3)	98.2 (97.9–98.6)		11.1 (12.7)
Timely antenatal care	48.9 (47.2–50.6)	68.4 (67.5–69.3)	73.7 (73.0–74.4)	88.2 (87.5–88.8)		39.3 (80.4)
Frequent antenatal care	70.3 (68.6–72.0)	88.8 (88.1–89.4)	92.7 (92.2–93.1)	93.1 (92.6–93.7)		22.8 (32.4)
Adequate antenatal care	76.7 (75.2–78.2)	80.4 (79.5–81.3)	86.1 (85.6–86.7)	87.8 (87.1–88.5)		11.1 (14.5)
Skilled delivery	85.5 (83.5–87.4)	95.4 (94.9–96.0)	96.3 (95.9–96.7)	97.1 (96.6–97.5)		11.6 (13.6)
Received postnatal care	60.0 (58.3–61.7)	83.1 (82.3–83.9)	82.5 (81.9–83.2)	81.7 (80.9–82.5)		21.7 (36.2)
Timely postnatal care	39.1 (37.7–40.5)	64.6 (63.6–65.5)	66.2 (65.5–67.0)	68.7 (67.7–69.7)		29.6 (75.7)
**Conditional **						
Received antenatal care	92.9 (92.0–93.9)	98.8 (98.6–99.0)	99.0 (98.9–99.2)	99.0 (98.8–99.2)		6.1 (6.6)
+ Skilled antenatal care	87.1 (85.6–88.6)	97.2 (96.8–97.6)	98.0 (97.8–98.3)	98.2 (97.9–98.6)		11.1 (12.7)
+ Timely antenatal care	47.3 (45.5–49.2)	67.8 (66.8–68.7)	73.2 (72.5–73.9)	87.6 (86.9–88.3)		40.3 (85.2)
+ Frequent antenatal care	43.2 (41.3–45.1)	64.9 (63.9–65.8)	71.4 (70.7–72.1)	84.9 (84.1–85.6)		41.7 (96.5)
+ Adequate antenatal care	38.0 (36.4–39.7)	55.4 (54.4–56.4)	63.2 (62.4–63.9)	76.2 (75.3–77.1)		38.2 (100.5)
+ Skilled delivery	36.7 (34.9–38.5)	54.7 (53.6–55.7)	62.2 (61.4–62.9)	75.0 (74.1–76.0)		38.3 (104.4)
+ Postnatal care	27.7 (26.1–29.2)	48.4 (47.4–49.4)	53.4 (52.5–54.2)	63.5 (62.5–64.5)		35.8 (129.2)
+ Timely postnatal care	17.8 (16.6–19.0)	38.1 (37.2–39.1)	43.1 (42.3–43.9)	53.3 (52.3–54.4)		35.5 (199.4)

CI: confidence interval.

^a^ *P* for trend < 0.001 for all independent and conditional coverage indicators.

In terms of conditional coverage indicators, we observed that the proportion of women receiving adequate antenatal care (defined as receiving timely, sufficient and appropriate care, delivered by skilled health personnel) doubled in the past 25 years, increasing from 38.0% (95% CI: 36.4–39.7) in 1994–1997 to 76.2% (95% CI: 75.3–77.1) in 2015–2018. In the 1994–1997 period, only 17.8% (95% CI: 16.6–19.0) of women receiving adequate antenatal care also received timely postnatal care; however, this proportion almost trebled over the 25-year period to 53.3% (95% CI: 52.3–54.4) in 2015–2018. We can also quantify the proportion who started to receive, but did not complete, the continuum of maternal health care during each period. For example, during 1994–1997 the proportion of women who received frequent antenatal care (defined as at least five antenatal consultations) was only 43.2% compared with the 92.9% who received at least one antenatal consultation (Table 2).

Our regression analysis showed that, compared with women aged 12–19 years, being in any of the older age groups (20–29, 30–39 and 40–54 years) was associated with a greater likelihood of receiving continuum of care coverage, after controlling for sociodemographic and obstetric characteristics (Table 3). Having social security and a level of education beyond elementary school is also associated with a greater likelihood of receiving continuum of care coverage. 

**Table 3:** Sociodemographic and obstetric characteristics affecting likelihood of receiving continuum of maternal health care, Mexico, 1994–2018

Sociodemographic and obstetric characteristics^a^	Adjusted odds ratio (95% CI)
**Period of last delivery**	
1994–1997	1.0 (–)
2004–2009	2.3 (2.2–2.5)
2010–2014	2.7 (2.5–3.0)
2015–2018	4.1 (3.8–4.5)
**Age at last delivery (years)**	
12 to 19	1.0 (–)
20 to 29	1.4 (1.3–1.5)
30 to 39	1.7 (1.6–1.8)
40 to 54	1.5 (1.3–1.7)
**Household head**	1.0 (0.9–1.1)
**Speaks at least one indigenous language**	0.7 (0.6–0.8)
**Marital status**	
Single	0.7 (0.7–0.8)
Married or cohabiting	1.0 (–)
Divorced, separated or widowed	0.8 (0.8–0.9)
**Education (years)**	
0–6 (none or elementary school)	1.0 (–)
7–9 (secondary school)	1.4 (1.3–1.5)
10–12 (high school)	1.6 (1.5–1.7)
13–24 (higher education)	2.1 (1.9–2.3)
**Employed in the last week**	1.1 (1.0–1.1)
**Health insurance**	
Social security	1.0 (–)
*Seguro Popular de Salud *	0.9 (0.8–0.9)
None	0.8 (0.7–0.8)
**Obstetric characteristics**	
Primiparous	1.2 (1.1–1.3)
History of stillbirth or infant death	0.9 (0.8–1.0)
At least one miscarriage or abortion	1.1 (1.0–1.2)
Health problem diagnosed during pregnancy	1.0 (0.9–1.0)
Health problem diagnosed during childbirth	1.0 (1.0–1.1)
**Socioeconomic status**	
Lowest	1.0 (–)
Low	1.6 (1.4–1.8)
Medium	1.8 (1.6–2.1)
High	2.2 (1.9–2.5)
Highest	2.4 (2.1–2.8)
**Area of residence^b^**	
Rural	1.0 (–)
Urban	1.0 (0.9–1.0)
Metropolitan	0.9 (0.9–1.0)

CI: confidence interval.

^a^ The data set includes women who have experienced at least one pregnancy.

^b^ We classified areas with less than 2500 inhabitants as rural, areas with 2500–100 000 inhabitants as urban and areas with more than 100 000 inhabitants as metropolitan.

**Fig. 1:**
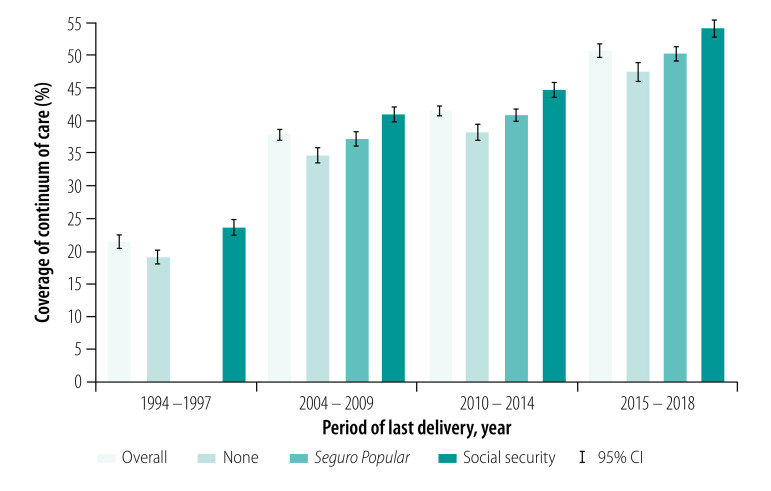
Continuum of maternal health-care coverage by health insurance status, Mexico, 1994–2018

In our temporal analysis, we observed an increase in national continuum of care coverage by around 30% from 1994–1997 to 2015–2018, regardless of health insurance status (Fig. 1). Assuming that most women covered by *Seguro Popular de Salud* were previously uninsured, Fig. 1 also highlights the large increase of 28.3% in the growth of continuum of care for uninsured women (from 19.0% to 47.3%) and of 31.1% for those changing from presumably no insurance to *Seguro Popular de Salud* (from 19.0% to 50.1%).

CI: confidence interval.

Note: The *Seguro Popular de Salud* programme was not introduced until 2003.

**Fig. 2:**
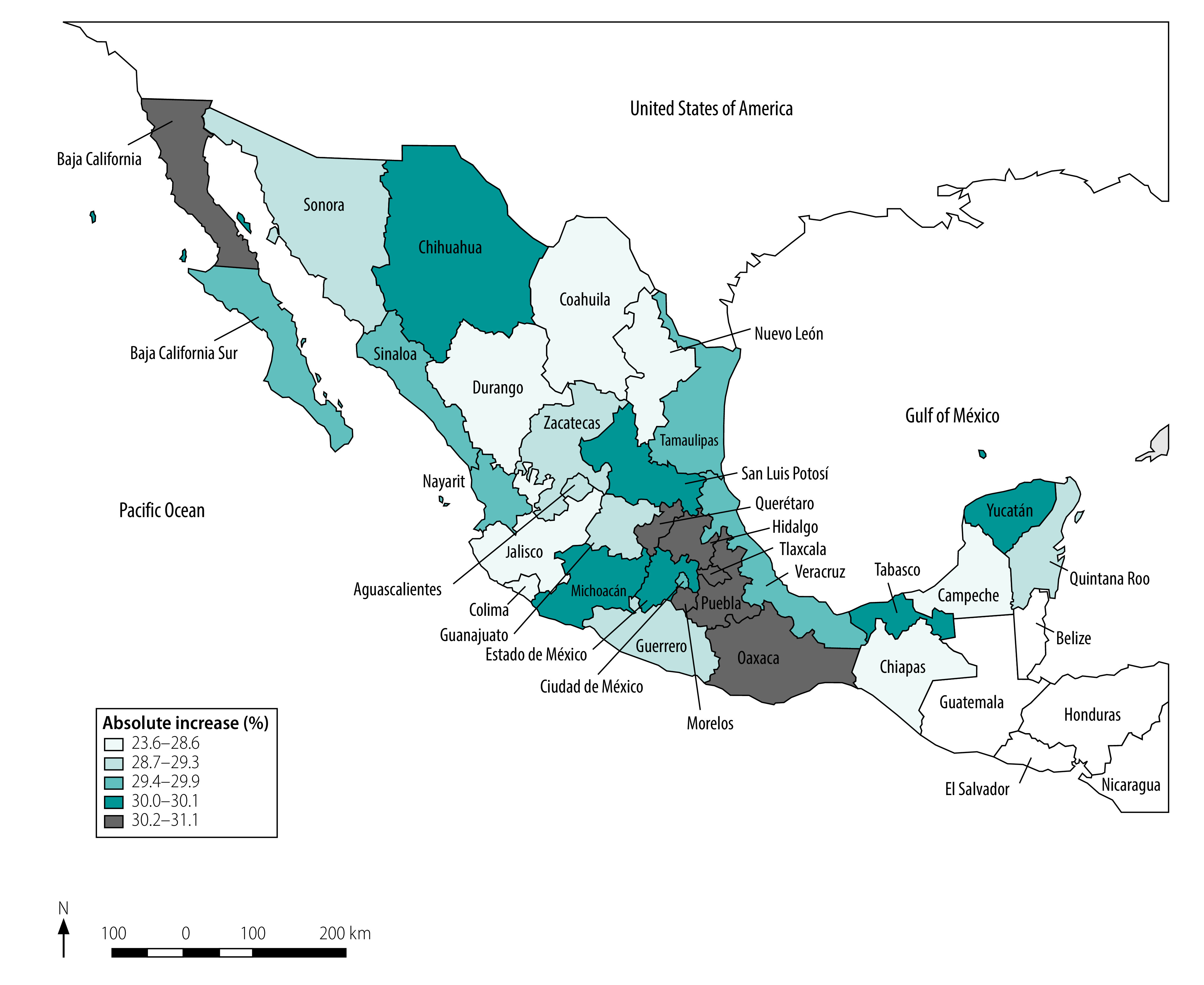
Quintiles of absolute increase in continuum of maternal health-care coverage by state, Mexico, 1994–2018

We mapped the wide geographical distribution of the quintiles of absolute increase in continuum of care coverage in Fig. 2. We list both absolute and relative increases in continuum of care coverage by Mexican state in Table 4, in which we observe the largest relative increases in the states of Chiapas (assigned a human development index, HDI, of low) and Durango (medium HDI).

Source of map: National Institute of Statistics and Geography, Mexico.

**Table 4:** Geographical analysis of women receiving continuum of maternal health care, Mexico, 1994–2018

State	HDI (2012)^a^	Estimated percentage of weighted population (95% CI)				Absolute (relative) increase in percentage from 1994–1997 to 2015–2018
		1994–1997 (*n* = 6 334 289)	2004–2009 (*n* = 8 424 843)	2010–2014 (*n* = 9 450 735)	2015–2018 (*n* = 5 612 585)	
Chiapas	Low	NA	25.3 (23.3–27.2)	28.3 (26.3–30.4)	36.6 (34.2–39.0)	23.6 (181.5)
Durango	Medium	16.5 (14.9–18.0)	30.7 (28.6–32.9)	34.1 (31.9–36.3)	43.0 (40.6–45.5)	26.5 (160.6)
Coahuila	Very high	17.3 (15.6–18.9)	32.0 (29.8–34.2)	35.4 (33.1–37.7)	44.4 (41.9–46.9)	27.1 (156.6)
Jalisco	High	17.7 (16.1–19.2)	32.5 (30.6–34.5)	36.0 (34.0–38.0)	45.1 (42.8–47.3)	27.4 (154.8)
Campeche	High	17.9 (16.3–19.5)	32.9 (30.8–34.9)	36.3 (34.2–38.5)	45.4 (43.1–47.8)	27.5 (153.6)
Colima	Very high	18.7 (17.1–20.3)	34.0 (32.0–36.1)	37.5 (35.4–39.7)	46.7 (44.4–49.0)	28.0 (149.7)
Nuevo León	Very high	19.7 (18.0–21.4)	35.5 (33.3–37.7)	39.1 (36.9–41.3)	48.3 (45.9–50.7)	28.6 (145.2)
Guanajuato	Low	19.9 (18.2–21.5)	35.7 (33.7–37.8)	39.3 (37.3–41.4)	48.6 (46.4–50.8)	28.7 (144.2)
Quintana Roo	High	19.9 (18.1–21.7)	35.8 (33.5–38.1)	39.4 (37.1–41.7)	48.6 (46.1–51.1)	28.7 (144.2)
Zacatecas	Low	20.1 (18.3–22.0)	36.1 (33.8–38.5)	39.8 (37.4–42.1)	49.0 (46.5–51.6)	28.9 (143.8)
Aguascalientes	High	20.2 (18.6–21.8)	36.2 (34.2–38.1)	39.8 (37.8–41.8)	49.1 (46.9–51.2)	28.9 (143.1)
Sonora	Very high	20.2 (18.3–22.1)	36.2 (33.9–38.6)	39.8 (37.4–42.2)	49.1 (46.5–51.7)	28.9 (143.1)
Guerrero	Low	21.2 (19.3–23.1)	37.6 (35.3–39.9)	41.3 (38.9–43.6)	50.6 (48.1–53.1)	29.4 (138.7)
Nayarit	Medium	21.3 (19.5–23.1)	37.7 (35.6–39.9)	41.4 (39.2–43.6)	50.7 (48.3–53.1)	29.4 (138.0)
Sinaloa	High	21.6 (19.7–23.6)	38.2 (35.8–40.6)	41.9 (39.4–44.4)	51.2 (48.6–53.8)	29.6 (137.0)
Baja California Sur	Very high	22.1 (20.2–23.9)	38.8 (36.5–41.0)	42.5 (40.2–44.7)	51.8 (49.4–54.2)	29.7 (134.4)
Ciudad de México	Very high	22.2 (20.3–24.1)	39.0 (36.8–41.2)	42.7 (40.4–45.0)	52.0 (49.6–54.5)	29.8 (134.2)
Veracruz	Low	22.3 (20.3–24.2)	39.1 (36.7–41.4)	42.8 (40.4–45.1)	52.1 (49.6–54.6)	29.8 (133.6)
Tamaulipas	High	22.4 (20.6–24.2)	39.3 (37.2–41.4)	43.0 (40.8–45.1)	52.3 (50.1–54.6)	29.9 (133.5)
Estado de México	High	22.8 (21.0–24.6)	39.8 (37.6–41.9)	43.5 (41.3–45.6)	52.8 (50.6–55.1)	30.0 (131.6)
Tabasco	Medium	22.8 (20.9–24.7)	39.8 (37.5–42.0)	43.5 (41.2–45.8)	52.8 (50.4–55.3)	30.0 (131.6)
Yucatán	Medium	22.8 (20.8–24.8)	39.8 (37.3–42.2)	43.5 (41.0–46.0)	52.9 (50.3–55.4)	30.1 (132.0)
Chihuahua	Medium	22.9 (21.0–24.8)	39.9 (37.6–42.2)	43.7 (41.3–46.0)	53.0 (50.6–55.5)	30.1 (131.4)
San Luis Potosí	Medium	23.0 (21.1–24.9)	40.0 (37.8–42.2)	43.7 (41.5–46.0)	53.1 (50.7–55.5)	30.1 (130.9)
Michoacán	Low	23.0 (21.1–24.9)	40.0 (37.8–42.3)	43.7 (41.5–46.0)	53.1 (50.8–55.5)	30.1 (130.9)
Puebla	Low	23.3 (21.4–25.1)	40.4 (38.3–42.5)	44.1 (42.1–46.2)	53.5 (51.3–55.7)	30.2 (129.6)
Tlaxcala	Medium	23.8 (22.0–25.5)	41.1 (39.1–43.1)	44.8 (42.8–46.8)	54.2 (52.0–56.3)	30.4 (127.7)
Oaxaca	Low	23.9 (21.7–26.1)	41.2 (38.7–43.8)	45.0 (42.5–47.5)	54.4 (51.7–57.0)	30.5 (127.6)
Morelos	High	24.6 (22.7–26.6)	42.2 (40.0–44.4)	46.0 (43.7–48.2)	55.3 (53.0–57.7)	30.7 (124.8)
Baja California	Very high	24.7 (22.7–26.8)	42.3 (39.9–44.7)	46.1 (43.7–48.5)	55.5 (53.0–57.9)	30.8 (124.7)
Querétaro	Very high	25.3 (23.3–27.3)	43.0 (40.8–45.2)	46.8 (44.6–49.0)	56.2 (53.9–58.5)	30.9 (122.1)
Hidalgo	Medium	26.1 (24.1–28.2)	44.1 (41.8–46.4)	47.9 (45.6–50.2)	57.2 (54.9–59.6)	31.1 (119.2)

CI: confidence interval; HDI: Human Development Index; NA: not applicable.

^a^ Source: *Programa de las Naciones Unidas para el Desarrollo*.^34^

## Discussion

This population-based study illustrates a notable improvement in the continuum of maternal health-care coverage over the last 25 years, with coverage more than doubling in all Mexican states. The data analysed also illustrate a social transformation in the living conditions of the female population in Mexico, with increased participation in higher levels of education and paid employment, and the greater economic independence these factors will bring. We observed an increase in the proportion of heads of households who are female, as well as in the single, divorced or widowed proportion of this population facing a pregnancy. This trend has also been accompanied by a decrease in total fertility rate, which is partly due to women’s greater engagement in reproductive decision-making.^35^^–^^37^

Our results showing the high numbers of women who begin but fail to complete the continuum of antenatal care are similar to a previous study, which showed that, although 84% of women received at least one antenatal care visit, only 38% received at least four visits.^7^ Similar levels of loss to follow-up in the continuum of care, associated with a higher risk of maternal and neonatal complications, have been reported elsewhere.^16^^,^^38^

In terms of the sociodemographic characteristics that increase the likelihood of receiving continuous care during pregnancy, childbirth and the puerperium, we observed that a higher level of educational attainment, recent employment and access to health insurance were all predictors. A study from Egypt showed similar results, and the authors suggested that educated women are more familiar with the meaning and importance of maternal health services, and may have better employment opportunities and a greater likelihood of access to medical insurance.^39^ As education level increases, the social gap between pregnant women and service providers decreases; women also become more aware of maternal health and experience an improved engagement with health-care services.^38^

Our calculation of the increasing proportion of women who received the continuum of care (from less than one fifth 25 years ago to over one half in 2018) highlights the achievements of Mexico’s maternal health-care policies. Since 1997, the anti-poverty programme *Prospera* (formerly *Progresa or Oportunidades*) has aimed to improve the provision and quality of basic social services, including reproductive health. Positive synergies between *Prospera* and *Seguro Popular de Salud* in the reduction of gaps in effective coverage for maternal health services have recently been assessed.^40^ Another successful health-care policy is the 2001 *Arranque Parejo en la Vida* programme that aimed to improve access to specialized delivery care, particularly in rural areas where the highest numbers of maternal mortality are reported. The government introduced the *Seguro Popular de Salud* in 2003 as part of the efforts to expand health coverage for those members of the population without social security;^22^^,^^41^^,^^42^ by 2018, about 45% of the Mexican population were covered by the *Seguro Popular de Salud*.^43^ This expansion in coverage financed the trebling of the health ministry budget from 2000 to 2018,^43^ allowing the provision of health-care services to be greatly enhanced.

Despite the significant progress achieved in maternal health care over the last 25 years in Mexico, important challenges remain. First, we documented a sustained and non-desirable increase in the proportion of deliveries by caesarean section over the study period. Our finding is consistent with a global increase in caesarean section deliveries, particularly in Latin America and the Caribbean.^44^^,^^45^ Although this issue in Mexico is beyond the scope of this study, some factors that may explain these results include the potential role of market forces, economic incentives and medico-legal issues in decision-making processes.^46^ Second, notwithstanding the increase in coverage in the continuum of maternal care seen with the introduction of the *Seguro Popular de Salud*, disparities remain between those with and without access to this programme. Third, improvements are needed in the efficiency and management of resources, the quality of services, the transparency of budgeting exercises and the expansion of existing coverage.^47^ This improvement is particularly important for the most vulnerable populations, such as indigenous women, those of lower socioeconomic status and adolescent women, for whom effective access to the continuum of care was the lowest.

Our study has several limitations. First, although the National Survey of Demographic Dynamics is a high-quality population-based survey, our analysis is subject to potential omitted variable bias, meaning that the conclusions reached here do not have the same strength as causal inference. Second, we used self-reported measures of outcome as variables and covariables; these may be subject to memory and interpretation bias, however, particularly for the timing of the initial prenatal visit variable. Third, the temporalities of our outcome and covariates were measured at the time of the survey and not at the time of the last delivery, meaning that some outcomes may have been subject to recall bias. Fourth, the surveys did not collect information about the exact locations at which prenatal, delivery and postpartum services were provided; it is therefore possible that women with social security may have received a particular type of health care at a facility not generally associated with this form of health care, potentially biasing our estimates of state-level continuum of care. Fifth, the changing definition of antenatal care as maternal health policies were updated and improved during our study period means that our antenatal care coverage figures for the earlier survey waves may be overestimated, indicating that our calculated increases in continuum of maternal health-care coverage over the 25-year period may be underestimated. Sixth, we have explored some objective indicators of quality of care focused on the care process, but have not provided evidence on subjective indicators of quality; however, prior studies have shown that large populations receiving reproductive health services in Mexico perceive that interactions with health personnel are inadequate.^48^^,^^49^ Regardless of the achievements depicted by our results, the ability to improve health care is also dependent upon the performance of health personnel. Finally, our data on postnatal care are limited. For instance, women may confuse a visit for their new infant with their own postpartum visit; in addition, we did not consider the satisfaction of care received at postpartum visits.

Despite demonstrating significant progress in continuum of care coverage over the last 25 years, important inequalities remain in the maternal health care received by indigenous and socioeconomically vulnerable women in Mexico. As well as government interventions to target the improvement of maternal health care received by these underserved populations, current and future health policies should aim to sustain the overall increase in continuum of care coverage. By prioritizing the continuum of care in research and health policy, we can reduce Mexico’s burden of disease, improve health outcomes and the quality of health care, and strengthen our health system, facilitating achievement of SDG 3, that is, ensuring healthy lives and promoting well-being for all at all ages.
